# Homologous recombination defects and how they affect replication fork maintenance

**DOI:** 10.3934/genet.2018.4.192

**Published:** 2019-04-03

**Authors:** Mi Young Son, Paul Hasty

**Affiliations:** 1 Department of Molecular Medicine and Institute of Biotechnology, UT Health San Antonio, 15355 Lambda Drive, San Antonio, USA; 2 The Mays Cancer Center, USA; 3 Sam and Ann Barshop Institute for Longevity and Aging Studies, USA

**Keywords:** homologous recombination, replication fork stability, RAD51 filaments, genomic integrity, gross chromosomal rearrangements

## Abstract

Homologous recombination (HR) repairs DNA double strand breaks (DSBs) and stabilizes replication forks (RFs). RAD51 is the recombinase for the HR pathway. To preserve genomic integrity, RAD51 forms a filament on the 3′ end of a DSB and on a single-stranded DNA (ssDNA) gap. But unregulated HR results in undesirable chromosomal rearrangements. This review describes the multiple mechanisms that regulate HR with a focus on those mechanisms that promote and contain RAD51 filaments to limit chromosomal rearrangements. If any of these pathways break down and HR becomes unregulated then disease, primarily cancer, can result.

## 1. Introduction

A cell might contain thousands of DNA lesions [1] that could potentially block DNA synthesis. These blocking lesions could stall or collapse RFs causing single-ended DSBs in DNA. HR repairs not only these breaks, but also stabilizes and restarts stalled RFs [2]–[6]. Central to HR is the recombinase RAD51. RAD51 forms a filament on ssDNA to protect it from degradation by MRE11 and other nucleases, but it also serves as the catalytic center for invasion and annealing to a homologous substrate usually provided by the sister chromatid. RAD51 function during HR can be divided into three phases: Presynapsis, synapsis and postsynapsis [7]. During the presynapsis phase, RAD51 is loaded onto ssDNA in a gap or at the 3′ end of a DSB. This process can remodel and remove toxic filaments. During the synapsis phase, RAD51 is involved in the invasion of a homologous double stranded DNA (dsDNA) template. During the postsynapsis phase, RAD51 is dissociated from the dsDNA to expose a 3′-OH that is needed for DNA synthesis. DSB repair involves the invading strand stabilizing a D-loop structure by capturing the complementary strand on the other DSB end to form of a double Holliday Junction (dHJ). The dHJ can be resolved to generate a crossover or a non-crossover product. For synthesis-dependent strand annealing (SDSA), the invading strand is displaced from the D-loop and anneals with its complementary strand in the gap or with the other DSB end. RAD51 promotes SDSA by blocking the formation of a dHJ [8]. The use of RAD51 is critical for repairing DSBs and for maintaining RF stability.

## 2. Interacting factors that facilitate and stabilize RAD51 nucleoprotein filaments

**Figure 1.**
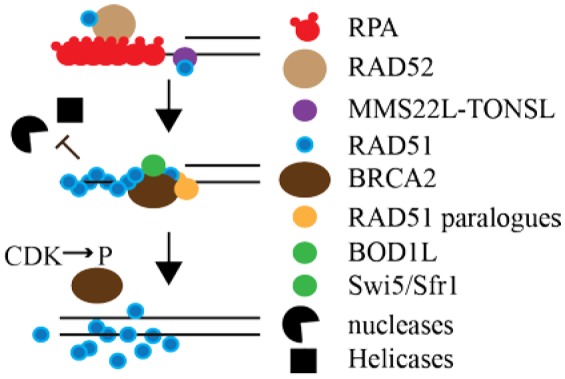
Model showing the dynamics of RAD51 filament assembly. RPA forms a filament on ssDNA with the aid of RAD52 that is also bound to RAD51. MMS22L-TONL binds to RAD51 and chromatin (histones not shown). BRCA2 along with the RAD51 paralogues, BOD1L and Swi/Sfr1 enable the formation of a RAD51 filament and stabilize the filament by suppressing helicases (BLM, FBH1) and nucleases (MRE11, DNA2) from unwinding and degrading the DNA strand. CDK phosphorylation of BRCA2 S3291 destabilizes the RAD51 filament.

RAD51 initiates HR by forming a filament on ssDNA via a self-interaction [9] that serves as the catalytic center for a homology search in the duplex and joint formation between homologous substrates (Figure 1). During presynaptic assembly, ssDNA is coated by replication protein A (RPA) [10]. RPA binds very tightly to ssDNA to minimize ssDNA secondary structure, prevent degradation and recruit DNA damage checkpoint kinases that initiate the DNA damage response [11]. RAD52 binds to RAD51 and to RPA-coated ssDNA and imparts an inhibitory effect on RPA turnover as viewed by single-molecule imaging and ssDNA curtains [12]. Yet, most of RPA and RAD52 were displaced from ssDNA due to the presence of RAD51. About 2–5 RAD51 monomers initiate nucleation and then additional RAD51 monomers bind to ssDNA [13],[14].

BRCA1 and BRCA2 suppress ovarian and breast cancer and both are critical for HR [15]. BRCA1 enables HR through 5′ to 3′ resection of DSBs to generate 3′ ssDNA overhangs that provide a substrate for RAD51 binding and by loading RAD51 onto the ssDNA [15]. BRCA1 colocalizes to sites of damaged DNA with the resection complex MRE11-RAD50-NBS1 (MRN) [16] and with the resection factor CtIP [17],[18]. BRCA1 enables BRCA2 recruitment to DSBs through the bridging protein PALB2 (partner and localizer of BRCA2) [19]–[22]. BRCA2 mediates replacing RPA with RAD51 by binding ATP-bound RAD51 to ss/dsDNA junctions and to ssDNA [23]–[28]. To facilitate RAD51 filament formation on ssDNA, BRCA2 associates with RAD51 through the eight BRC motifs encoded by exon 11 [29],[30]. The BRC motifs recruit RAD51 to the site of DNA damage [31]–[33] by interacting with RAD51 monomers and nucleoprotein filaments to nucleate RAD51 specifically onto RPA-coated ssDNA. BRCA2 BRC motifs interact with RAD51 to reduce the binding of RAD51 to duplex DNA and stimulate RAD51-mediated DNA strand exchange [26]. BRC1-4 motifs bind to free RAD51 and reduce its ssDNA-dependent ATPase activity while preventing it from binding to dsDNA [32],[34]. BRC5-8 motifs couple with the BRCA2 DNA binding domain enable RAD51 pairing to DNA that complements those of BRC1-4. BRC5-8 repairs nuclease-induced DSBs and accelerates the assembly of RAD51 complexes to damaged DNA [35]. Thus, the RAD51-BRC motifs interaction is necessary for replacing RPA with RAD51.

In addition to the BRC motifs, RAD51 associates with BRCA2 in a region encoded by exon 27 (Ex27) [36],[37]. Ex27 does not bind to RAD51 monomers, but instead binds an interface created by two adjacent RAD51 protomers in the RAD51 filament to stabilize the filament from BRC-mediated dissociation [38],[39]. Ex27 interacts with oligomerized RAD51 to stabilize the filament. Ex27 disassembles the nucleoprotein filament at the G_2_-M transition after CDK phosphorylation on BRCA2 S3291 (S3215 in mouse). Furthermore, the Ex27 domain is needed to block MRE11-degradation at stalled RFs but does not enable DSB repair [40]. BRCA2 C-terminal mutants cause rapid foci disassembly and mitotic entry [41]. Mice and cells that express BRCA2 deleted for the exon 27 encoded region exhibit hypersensitivity to γ-radiation, premature replicative senescence, chromosomal instability, increase in the levels of stalled RFs and a reduction in survival due to an early onset of cancer [4],[42],[43]. The BRC motifs and Ex27 are required for DSB repair and RF maintenance, respectively [40].

The RAD51 paralogs are another set of factors that regulate the formation of RAD51 nucleoprotein filaments on ssDNA and catalyze the strand invasion with homologous duplex DNA. In *Caenorhabditis elegans*, the RAD51 paralogs RFS-1/RIP-1 remodel and stabilize RAD-51 filaments [44]. RFS-1/RIP-1 prevents RAD-51 dissociation from ssDNA by capping the 5′ end of RAD-51-ssDNA filaments. Nucleotide binding, but not hydrolysis, is required by RFS-1/RIP-1 to stabilize the filament [45]. The mammalian RAD51 paralogs have 20–30% identity with RAD51 and include RAD51B, RAD51C, RAD51D, XRCC2 and XRCC3. They form two complexes: RAD51B/RAD51C/RAD51D/XRCC2 (BCDX2) and RAD51C/XRCC3 (CX3) [46]. In response to damaged DNA, the BCDX2 and CX3 complexes function at different stages during HR [47]. BCDX2 functions after BRCA2 foci formation but before RAD51 foci formation while CX3 works after RAD51 foci formation. The BCDX2 complex is epistatic to BRCA2 and has the most affinity for branched DNA substrates and likely functions in the formation and stabilization of RAD51 filaments [46]. The CX3 complex resolves HJ. The BC subcomplex shows RAD51 mediator activity [48] while the CX3 complex and the DX2 subcomplex shows strand exchange activity [49],[50]. The RAD51 paralog protein complex is modified by the RNF138 ubiquitin ligase that degrades RAD51D and enhances the interaction between RAD51D and XRCC2. RNF138 depletion reduces RAD51 foci, enhances sensitivity to genotoxins and increases chromosomal instability [51]. These results suggest that RAD51 paralogs facilitate BRCA2/RAD51-mediated HR at different stages.

There are a variety of other factors that stabilize RAD51 filaments. The MMS22L-TONSL complex is apart of the response to replication stress and is needed for the efficient formation of RAD51 foci after DNA damage. MMS22L-TONSL associated with RPA-coated ssDNA and MMS22L directly interacts with RAD51 to stimulate RAD51-ssDNA filament formation and RAD51-dependent strand exchange activity, but limits RAD51 assembly onto dsDNA [52]. MMS22L-TONSL recruitment of RAD51 is dependent on chromatin assembly [53]. MMS22L-TONSL accumulates at stressed RFs and depletion of MMS22L-TONSL impairs HR and increases sensitivity to type 1 topoisomerase inhibitors. The levels of DNA DSBs increases due to a failure to complete DNA synthesis after fork collapse. In depleted cells, camptothecin (CPT)-induced DSBs are resected normally, but RAD51 loading is defective [54],[55]. CPT is a type I topoisomerase inhibitor that causes breaks at RFs [4]. BOD1L is another protein that stabilizes RAD51 and its removal causes extensive nascent strand degradation [56]. Inhibiting DNA2-dependent resection or knocking down the BLM and FBH1 helicases in BOD1L-deficeint cells suppresses this phenotype indicating BOD1L as a genome integrity regulator that suppresses nucleolytic degradation of stressed RFs. RAD51AP1 binds selectively to branched DNA structures during joint molecule formation and interacts with RAD51 to stabilize RAD51-mediated D-loops [57],[58]. Swi5 and Sfr1 form a heterodimeric complex that associates with RAD51 and prevents RAD51 dissociation to stabilize RAD51 filaments [59]. These are examples of the complexity of RAD51 regulation and there are more proteins shown to interact with RAD51 but their function and role are not well defined.

## 3. ATP binding and hydrolysis are essential for RAD51 nucleoprotein filament formation and stability

RAD51 nucleoprotein filament formation requires ATP binding and hydrolysis and it contains a Walker A motif with a highly conserved lysine (K133) that is important for ATPase activity. Mutations in the Walker A motif help define its function with K133A (KA) and K133R (KR) prohibiting ATP binding or ATP hydrolysis, respectively [60]. ATP binding is important for assembly and stabilization of a catalytically active filament, while ATP hydrolysis is important for its disassembly and release from DNA [61]; yet, neither of these mutants impact the equilibrium binding affinity of RAD51 to ssDNA and RAD51-protein interactions [60]. Unlike K133R, the K133A mutant induces topological changes in duplex DNA in an ATP-dependent manner. Thus, K133A is more severe biochemically than K133R. A biological analysis showed that, expression of either K133A or K133R causes RF stalling [4] and failed DSB repair [62]. These cells exhibited gross chromosomal rearrangements that included multiple breakpoints within the same chromosome and they were hypersensitive to CPT. RAD51 K133A/R conjugated to eGFP was located to chromatin at the same level as wildtype (WT) RAD51-eGFP but K133A/R-eGFP was not located to stressed RFs or CPT-induced foci at WT-eGFP levels; thus accounting for the RF defects and the chromosomal aberrations.

ATP-bound RAD51 filaments define two distinct conformation states (compact and extended) and upon hydrolysis an ADP-bound RAD51 filament can disassociate from ssDNA [63], just as it does from dsDNA [64]. Using electron microscopy (EM) the ATP-bound RAD51 filaments display an extended architecture while ADP-bound filaments display a comparatively compact architecture relative to B-DNA [65]. Using electron cryo-microscopy the structure of the human RAD51 presynaptic filament is measured at 3.5–5.0A resolution with RAD51 encasing ssDNA with a 103A pitch for the helical filament, comprising 6.4 protomers per turn with a rise of 16.1A and a twist of 56.2A. [66]. Wildtype RAD51 filaments have an open conformation compared to the K133-mutants due to differences in helical pitch. A series of isomerization or dissociation events mediated by the nucleotide binding state is important for RAD51 nucleoprotein filament dynamics. Compared to RAD51 WT with ATP using EM, K133A and K133R mutants with ATP and RAD51 WT with AMP-PNP reveals a closed structure upon 3D reconstruction with a pitch of 108 and 71–80, respectively [67]. Using an electrophoretic mobility shift assay (EMSA), WT forms faster migrating complexes compared to K133R and K133A that could reflect their different conformations. When exposed to DNase1 or S1 nucleases, WT filaments exhibits smaller DNA protection compared to K133R and K133A or WT exposed to AMP-PNP suggesting the lower helical pitch is a steric obstruction to these nucleases. Stopped-flow experiments suggests that RAD51 filaments formed in the presence of ATP undergo two consecutive isomerization reactions after the initially binding to ATP, one of these isomerization steps is driven by ATP hydrolysis. The β-isoform of BCCIP (BRCA2 and CDKN1A Interacting Protein) interacts with RAD51 to promote ADP release and stimulate an active presynaptic filament [68]. These results suggest that filament assembly and disassembly can be regulated by nucleotide co-factors, but also indicate possible regulatory points by RAD51 accessory proteins and point to the need for positive and negative regulators.

## 4. Post-translational modifications that regulate RAD51 function

RAD51 undergoes multiple post-translational modifications. Often these modifications involve kinases. c-ABL phosphorylates RAD51 Y54 and Y315 with phosphorylation of Y315 occurring before Y54 [69]. The Philadelphia chromosome is a fusion of BCR to c-ABL that produces a protein with unregulated tyrosine kinase activity [70]. BCR-ABL influences cell cycle [71] and DNA damage responses [72] to increase the incidence of chronic myelogenous leukemia (CML) and acute lymphoblastic leukemia (ALL). BCR-ABL's constitutive phosphorylation of RAD51 Y315 might cause homologous recombination between similar but not identical repeats (called homeologous recombination) that can lead to chromosomal instability with cancer relapse and progression [73]. Furthermore, phosphorylation of RAD51 Y315 was required for oligomerization-defective RAD51 mutants to associate with chromatin [74]. c-ABL phosphorylation of RAD51 Y54 inhibits RAD51 binding to DNA and ATP-dependent DNA strand-exchange [75]. Y54 phosphorylation enhanced RAD51 recombinase activity by modifying the formation of the filament, and allowing RAD51 to compete with RPA while Y315 phosphorylation did not impact these activities [76]. Substituting tyrosine with glutamic acid mimics the negative charge after phosphorylation. RAD51 Y54E, RAD51 Y315E and RAD51 Y54E/Y315E exhibit loss of RAD51 oligomerization and strand exchange activity [77]. In addition, RAD51 interacts with checkpoint kinase (CHK1) and is phosphorylated on T309 in a CHK1-dependent manner. After exposure to hydroxyurea, RAD51 nuclear foci failed to form after knockdown of CHK1 and expression of RAD51 T309A caused hypersensitivity to hydroxyurea, a ribonucleotide reductase inhibitor that stresses RFs [78]. Furthermore, Polo-like kinase 1 (PLK1) phosphorylates RAD51 S14 and casein kinase 2 (CK2) phosphorylation of RAD51 T13 that initiated binding to the Nijmegen breakage syndrome (NBS) protein to facilitate RAD51 binding to damaged sites [79]. Thus, posttranslational modifications are essential for regulating RAD51 and HR.

## 5. Interacting factors that destabilize RAD51 nucleoprotein filament formation

There are three basic reasons to destabilize RAD51 filaments [7]: (1) Toxic RAD51 filaments need to be removed to prevent potential chromosomal rearrangements and deletions especially when the strand anneals to a nonallelic repeat [80]. (2) SDSA pathway is selected over the cross-over mechanism. (3) RAD51 is removed from post-synaptic filaments. To negatively regulate RAD51 filaments, helicases and translocases (RECQ5, FBH1, RAD54, RTEL1, HELQ, BLM) dismantle or destabilize RAD51 intermediates [7],[81]. RECQ5 [82] and FANCJ [83] are helicases that dismantle RAD51 filaments from ssDNA that enable MUS81-mediated cleavage [84], while BLM dissolves presynaptic filaments [85] and promotes dissolution of hemicatenanes following branch migration between two HJs to produce non-crossovers [86]. FANCM is a translocase that unwinds RAD51 filaments from the D-loop intermediates and promotes branch migration of HJs [87]. Proliferating cell nuclear antigen (PCNA) is a DNA polymerase clamp that is critical for DNA synthesis and the PCNA-associated recombination inhibitor (PARI) inhibits HR after RF stalling and during DSB repair [88]–[90]. PARI suppresses recombination by interacting with SUMOylated PCNA and RAD51 [89]. SUMOylated PCNA interaction with PARI negatively regulates DNA extension by polymerases [90].

## 6. Consequences of RAD51 variants and altered RAD51 levels

An accumulation of genetic mutations can contribute to cancer development, progression and metastasis [91]. HR was first identified as a tumor suppressor pathway when RAD51 was found to associate with BRCA1 [92] and BRCA2 [36],[93]. Women with a heterozygous mutation for BRCA2 are susceptible to breast and ovarian cancer [94],[95]. Loss of heterozygosity (LOH) precedes cancer development such that only the tumor is null for BRCA2. In addition, heterozygous carriers of a BRCA2 mutation in males show a significant increase in a variety of cancers [96]. However, not many RAD51 mutations have been found in the human population, likely because it's function is essential for cellular proliferation [97]. Yet, there two mutations that cause Fanconi anemia (FA): T131P and A293T. FA is an autosomal recessive genetic disorder that shows developmental problems and chromosomal instability leading to bone marrow failure and increased cancer predisposition [98]. FA-associated RAD51 mutations disrupt the Walker A motif causing aberrant ATP binding and hydrolysis leading to failed filament assembly and reduced RF protection [99]. RAD51 T131P is defective in RF protection independent of its DNA strand exchange activity. Cells expressing RAD51 T131P are capable of HR-mediated DSB repair but exhibit sensitivity to mitomycin c (MMC) and MMC-induced chromosomal abnormalities [100]. MMC is a crosslinking agent to causes DSBs at RFs [101],[102]. The A293T mutation acts as a dominant negative and it interferes with RAD51 WT binding to DNA, affected D-loop formation, ATP hydrolysis, DNA binding and filament formation [103]. T131P and A293T destabilize RFs [99]. There are other reports of RAD51 SNPs that associate with cancer. These include S121Y, E169Q, S296L, R150Q, Q268P and Q272L (https://cancer.sanger.ac.uk/cosmic, http://www.cbioportal.org/index.do). Q268P and Q272L are associated with lung and kidney tumors and these mutations are located in the DNA binding loop 2 region that is important for DNA binding and ATP hydrolysis and DNA strand exchange [104]. Both mutants when mixed with wild type RAD51 exhibit reduced DNA strand exchange activity. Q268P and Q272L are two RAD51 variants found to associate with lung and kidney tumors in humans [104]. R150Q is associated with familial breast cancer [105] and G135C is associated esophageal cancer [106] and breast cancer [107] while RAD51 G127T might protect against head and neck cancer [107]. Biochemical and biological analyses were not performed for most of these variants so we do not know if the have an impact on RAD51 filament dynamics.

The expression level and localization of RAD51 are important to maintain genomic stability. Human RAD51 is often overexpressed in tumors causing resistance to chemotherapeutics and increasing genomic instability that further contributes to cancer etiology [108] while RAD51 haploinsufficiency can cause congenital mirror movements (involuntary movements on one body side that mirror intentional movements on the other body side) describing a role for RAD51 in neurodevelopment [109]. RAD51 localization can also play an important role in maintaining genomic stability. RAD51 does not contain a NLS but RAD51 can be imported into the nucleus by binding to RAD51C since it contains a nuclear localization signal [110]. RAD51 can be exported into the cytoplasm through its nuclear export signal (NES). A BRCA2 variant (D2723H) affects the nuclear localization of BRCA2 and by association, RAD51. In turn, BRCA2 D2723H is localized to the cytoplasm to inhibit the RAD51's nuclear retention by exposing the NES that would otherwise be obscured by the BRCA2-RAD51 interaction. Thus, NES-masking interactions localize BRCA2 and RAD51 in the nucleus [111]. hCAS/CSE1L (cellular apoptosis susceptibility/chromosome segregation 1-like) transports importin α from the nucleus to the cytoplasm suggesting that hCAS/CSE1L performs the nuclear-cytoplasmic shuttling [112]. hCAS/CSE1L negatively regulates RAD51 protein levels and RAD51 foci formation in response to DNA damage through a direct interaction with RAD51 [113]. Karyopherin α2 (KPN2) shuttles proteins between the cytoplasm and nucleus and a high level of KPNA2 expression was associated with subcellular localization of RAD51 and other proteins important for HR like BRCA1 to the cytoplasm and a low level of nuclear expression. A high level of KPNA2 expression associates with a poor prognosis for breast cancer [114]. Cofilin is an actin-associated protein that regulates actin dynamics. Under stress, cofilin imports actin monomers into the nucleus. Cofilin overexpression increases ionizing radiation (IR)-induced sensitivity in human non-small lung cancer cells with lower levels of RAD51 and other DNA repair proteins [115].

P53 is a transcriptional regulator that responds to a variety of stressors including DNA damage that ultimately suppresses oncogenesis in over half of all cancers [116]. DNA damage activated p53 through the ATM (ataxia telangiectasia mutated) protein kinase. p53 represses HR [117],[118] likely through its direct interaction with RAD51 [119],[120] and with heteroduplex joints [121]. p53 reduces RAD51-mediated strand exchange [122]. RAD51 interacted to a lesser extend with these p53 variants (135Y, 249S and 273H) [123]. The p53 273H variant is associated with cancer [124] and is defective in reducing recombination [125]. Association with RAD51 is confirmed to be the foundation of p53's anti-recombinogenic activity when expressing RAD51 186P, a mutant that cannot bind to p53 [119]. The reason for p53's anti-recombinogenic function might be to suppress homeologous recombination [125] perhaps complimentary or in synergy with MSH2, a member of mismatch repair that is well know for prohibiting homeologous recombination by its action on mismatched heteroduplexes [126].

## 7. Factors that degrade the nascent strand in the absence of stabilized RAD51 filaments

Regulation of RAD51 filament assembly is key to ensure efficient HR. RAD51 protects the nascent strand from degradation by MRE11 and other nucleases at and behind the RF [127]. BRCA2-mediated binding of RAD51 to form a filament on replicating DNA suppresses the formation of single-stranded gaps and Pol α binds directly to RAD51 to minimize ssDNA gaps [128]. Stable RAD51 filaments suppress MRE11-mediated nascent strand degradation in BRCA2-deficient cells [40],[129],[130] that likely causes the lethality observed in cells deleted for either BRCA2 [131] or RAD51 [97]. The resected arms provide a substrate for MUS81 cleavage that promotes POLD3-dependent rescue [129]. PTIP (Pax2 transactivation domain-interacting protein) is involved in HR [132] and it contributes to degradation of the nascent strand. A PTIP deficiency restores reversed fork frequency and chromosomal integrity [40],[128],[133],[134]. Loss of PTIP inhibits MRE11 recruitment to stalled RFs and allows BRCA2-deleted cells to survive [135],[136]. In addition, RADX antagonizes RAD51 to destabilize RFs especially in cells defective for BRCA2, BOD1L and FA [137]. RADX is an RPA-like ssDNA-binding factor that is recruited to sites of replication stress [138],[139]. Removing RADX stabilizes these RFs by preventing MRE11- and DNA2-mediated nascent strand degradation while overexpressing RADX degrades RFs. RADX competes with RAD51 for binding to ssDNA implicating a role for RADX monitoring RAD51 levels for RF reversal and protection. This is important since RAD51 levels are critical for these activities [137]. Furthermore, in *BRCA2*-mutant cells, deficiency of the nucleosome remodeling factor CHD4 will lead to RF protection and resistance to chemotherapy. This resistance occurred due to RF protection and reversal; thus depriving a substrate for MRE11-mediated degradation as opposed to restoration of HR [135].

Deficient levels of BRCA2 and RAD51 allow MRE11 or DNA2 degradation to occur on the nascent strand that is dependent on replication fork remodelers SMARCAL1 (SWI/SNF-related matrix-associated actin-dependent regulator of chromatin subfamily A-like protein 1), ZRANB3 (Zinc Finger Ran-binding Domain-containing Protein 3) and HLTF (helicase-like transcription factor) [128],[140]. SMARCAL1, ZRANB and HLTF are members of the SWI/SNF family of proteins that have helicase and ATPase activity. These proteins employ a remodeling mechanism to stabilize stressed RFs to bypass damaged templates. In BRCA2-deficienct cells treated with replication stress-inducing agents, SMARCAL1, HLTF and ZRANB3 translocase activities were required for fork regression and subsequent MRE11-mediated nascent strand degradation and its depletion restored RFs and genomic integrity [128],[140],[141].

SMARCAL1 is recruited to stalled RF sites through an RPA interaction [142]–[146]. SMARCAL1 moves with the elongating RF, and converts RPA-bound ssDNA into dsDNA [147]. It can bind three-way and four-way HJs and model RFs without a designed ssDNA region. Furthermore, SMARCAL1 promotes branch migration and fork regression to remodel these DNA substrates implying that it surveys RFs for damage and if present it remodels the fork to enable repair and restart [148]. SMARCAL1-deficiency causes MUS81-dependent DSBs and hypersensitivity to agents that induce replication stress [142].

HLTF is recruited to stalled RFs and it has HIRAN, RING, and SWI/SNF domains that enable DNA-binding, PCNA-polyubiquitin-ligase, and dsDNA-translocase activities, respectively. The HIRAN domain recruits HLTF to a stalled RF and orients the direction for dsDNA translocase motor domain for fork reversal [149]–[153]. HLTF also polyubiquitinates PCNA K164 [154] and is a functional mammalian ortholog to yeast Rad5, a member of DNA damage tolerance (DDT) that is essential for PCNA polyubiquitination [155]. DDT bypasses lesions that obstruct RF progression. Initially discovered in *Saccharomyces cerevisiae*, DDT maintains RFs through two branches: (1) translesion synthesis (TLS) and (2) template switch (TS) [156],[157]. Both are controlled by ubiquitinating PCNA. RAD6/RAD18 monoubiquitinate PCNA K164 to induce TLS by switching pol δ/ε with a TLS polymerase while UBC13/MMS/RAD5 polyubiquitinate PCNA K164 to induce TS by means that are poorly understood. These pathways stabilize RFs but have the potential to cause mutations since some of the TLS polymerases have low stringency [158] and since TS can rearrange chromosomes when the template strand anneals to a nonallelic repeat [80].

ZRANB3 is recruited to stalled RFs through polyubiquitinated PCNA K164. ZRANB3 has a substrate recognition domain that is necessary to recognize forked DNA structures, hydrolyze ATP, catalyze fork remodeling, and act as a structure-specific endonuclease [159]. ZRANB3 localizes to sites of replication stress and interacts with PCNA K63-linked polyubiquitin chains to affect fork reversal [141],[160].

## 8. A model for the generation of genomic instability associated with an HR-defect

**Figure 2.**
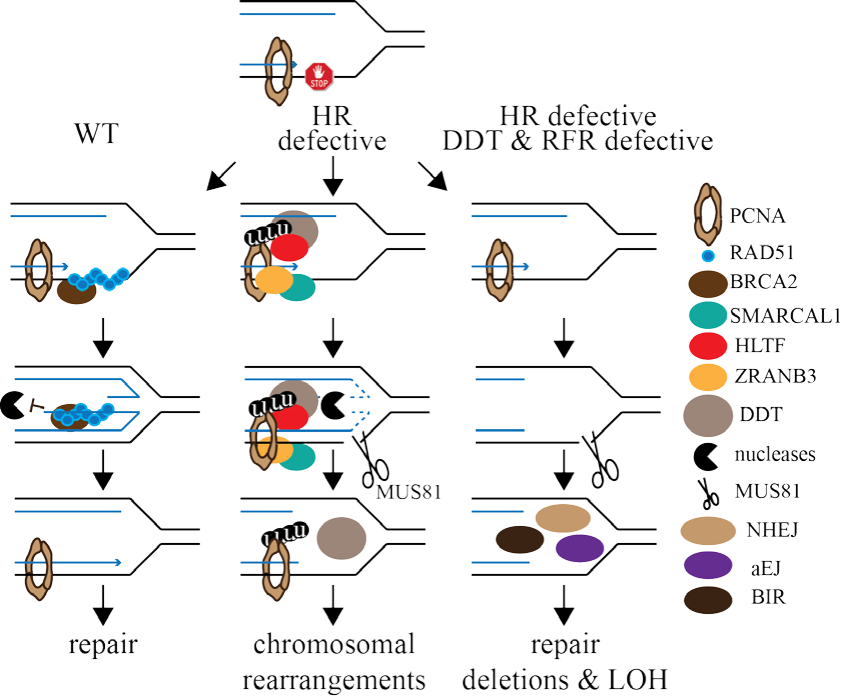
Three potential outcomes for RF maintenance and DSB repair that implicate a genetic interaction between HR and DDT that includes the replication fork remodelers (RFRs): SMARCAL1, HLTF and ZRANB3.

To preserve chromosomal integrity, we propose a model for RAD51 and RF remodelers (RFRs: SMARCAL1, HLTF and ZRANB3). In our model we propose that RFRs are apart of the DDT pathway. To support our assertion, HLTF is a functional Rad5 homolog and it interacts with UBC13 and PCNA [154]. HLTF promotes PCNA K164 (K63-linked) polyubiquitination and HLTF-deleted cells exhibit elevated chromosome breaks and fusions after methyl methane sulfonate treatment. Therefore, HLTF participates in PCNA polyubiquitination. Furthermore, ZRANB3 associates with polyubiquitinated PCNA K164 [141],[160]. It is possible that SMARCAL1 is a member of DDT due to its similar phenotype with ZRANB3 and HLTF. There are three scenarios for stalled RFs (Figure 2). First, wild type conditions result in repaired DSBs and stable RFs with few chromosomal rearrangements. DDT would not be typically used under these conditions. Second, defective HR causes RF reversal that is associated with excessive MRE11-mediated degradation of the nascent strand that could induce MUS81 to generate a DSB. The template switch branch of DDT will correct the damage but with an increase in chromosomal rearrangements. At this time we do not know if RAD51 is apart of DDT and a RAD51 defect could alter DDT function increasing its mutagenicity. Third, defective HR and DDT result in unrepaired DSBs without RF reversal. HR is the primary pathway that repairs DSBs during DNA synthesis; yet, a deficiency of the RFRs leads to activation of an alternative repair mechanism that depends on MUS81-catalyzed cleavage of the damaged fork. These pathways include nonhomologous end joining (NHEJ), alternative end joining (aEJ), single strand annealing (SSA) and break induced replication (BIR). NHEJ repairs DNA DSBs by joining the ends together without a template and is primarily used in G_1_ phase but can also be used in S phase [161]. The aEJ mechanism is a minor pathway compared to HR and NHEJ in normal cells but it can be used in cancer cells to generate genomic rearrangements [162]. One form of aEJ is SSA that repairs DSBs by annealing of complementary single strands between two direct repeats that were exposed by exonucleases. This annealed intermediate is processed by nucleases removing the single strand tails to generate a deletion [1]. BIR repairs DSBs with one free end by strand invasion into a homologous duplex DNA and replicating the entire chromosome end causing LOH [163]. In the absence of fully efficient HR and DDT any one of these pathways could repair a DSB at a RF with the risk of generating chromosomal abnormalities.

(1) HR and DDT/RFRs are both WT. Refer to figure 1 for the formation of a RAD51 filament. HR efficiently repairs a DSB and stabilizes the RF and few chromosomal rearrangements. DDT is not typically used in these cases.
(2) DDT/RFRs are WT, but HR is defective. DDT is composed of RAD6/RAD18 and UBC13/MMS/HLTF (RAD5 orthologue). SMARCAL1 is recruited to stalled RF sites through an RPA interaction. HLTF polyubiquitinates PCNA K164. ZRANB3 is recruited to stalled RFs through polyubiquitinated PCNA K164. These RFRs induce RF regression but without a stabile RAD51 filament to protect the nascent strand from MRE11/DNA2-mediated degradation. MUS81 could generate a DSB and the template switch branch of DDT could correct the problem with a greater risk to rearranging chromosomes.
(3) Both HR and DDT/RFRs are deficient so there is no RF reversal. MUS81 could generate a DSB that will be repaired by another DSB repair pathway. There could be deletions or LOH depending on the pathway used to repair the DSB.

## 9. Potential anti-cancer therapeutics that target HR and RAD51

Defects in the HR pathway offer an opportunity to treat cancer. PARP1 [Poly(ADP-ribose) polymerase 1] inhibitors are currently being used in HR-deficient tumors [164]. PARP1 repairs single strand breaks (SSBs) and inhibiting PARP1 results in more SSB lesions that become DSBs that are normally repaired by HR [165]. For a patient with breast cancer that is caused by an inherited heterozygous BRCA2 mutation, only the tumor cells are mutant for BRCA2 due to LOH. Thus, the tumor is more susceptible to PARP1 inhibitors than the patient. PARP1 inhibitors could also be used in combination with clastogenic agents to improve their effect [166]. RAD51 inhibitors show promise for treating cancers, especially for those that exhibit elevated levels of RAD51 expression. Elevated levels of RAD51 are associated with breast cancer and chronic myeloid leukemia (CML). Furthermore, elevated levels of RAD51 are associated with resistance. A small molecule, IBR2, inhibits the formation of RAD51 multimers and results in increased proteasome-mediated RAD51 protein degradation, reduced IR-induced RAD51 foci and HR. Exposure to IBR2 inhibits cancer cell growth and induces apoptosis. Furthermore, IBR2 improves survival when given to a mouse model with drug-resistant CML and inhibits the proliferation of CD34+ progenitor cells derived from CML patients [167]. Another small molecule that attenuates RAD51 function is T0070907, a PPARγ inhibitor. PPARγ influences RAD51 and exposing cells to T0070907 attenuate RAD51 levels and ionizing radiation-induced foci causing centrosome amplification and multipolar mitotic spindle formation in cervical cancer cells but not in wildtype cells [168]. Therefore, these inhibitors might kill only cancer cells. Furthermore, our knowledge about RAD51 and the factors that modify its action can be exploited for cancer therapy. Generation of inhibitors to proteins like SMARCAL1 and ZRANB3 could be beneficial for treating cancer with defects in HR since their knockdown reduced the level of chromosomal instability in BRCA2-deficient cells exposed to clastogenic agents [140]. Chromosomal instability enhances resistance and metastasis. Therefore, drugs directed against these new targets could be used to complement the current treatment of HR-deficient cancers.

## 10. Conclusions

HR repairs DNA DSBs and stabilizes RFs to maintain faithful DNA synthesis. The RAD51 recombinase is a critical component of the HR pathway that forms a filament on ssDNA to repair DSBs and protect RFs. A multitude of proteins regulate the formation of a RAD51 filament. Positive regulators like RAD52, BRCA1/2, MMS22L-TONL, RAD51 paralogues, BOD1L and Swi/Sfr1 enable the formation and stability of RAD51 filaments by suppressing helicases and nucleases from unwinding and degrading the DNA strand. In addition to these helicases and nucleases, negative regulators of RAD51 filament formation and stability include proteins like RADX. The dynamics of RAD51 filament formation and stability is important for tumor suppression by maintaining genomic stability. With a defect in the formation and stability of these filaments, the RFs reverse to stabilize the fork. But this could enable DDT to take over; thus causing GCRs. Anti-cancer therapeutics can exploit defects in HR to enhance the killing of tumor cells and perhaps novel drugs can be developed that disable DDT to reduce the level of genomic instability that would otherwise lead to resistance and metastasis.
